# Studies in RF Power Communication, SAR, and Temperature Elevation in Wireless Implantable Neural Interfaces

**DOI:** 10.1371/journal.pone.0077759

**Published:** 2013-11-06

**Authors:** Yujuan Zhao, Lin Tang, Robert Rennaker, Chris Hutchens, Tamer S. Ibrahim

**Affiliations:** 1 Department of Bioengineering, University of Pittsburgh, Pittsburgh, Pennsylvania, United States of America; 2 School of Electrical and Computer Engineering, The University of Oklahoma, Norman, Oklahoma, United States of America; 3 Behavioral and Brain Sciences, Erik Jonsson School of Engineering, University of Texas Dallas, Richardson, Texas, United States of America; 4 School of Electrical and Computer Engineering, Oklahoma State University, Stillwater, Oklahoma, United States of America; 5 Department of Radiology, University of Pittsburgh, Pittsburgh, Pennsylvania, United States of America; Glasgow University, United Kingdom

## Abstract

Implantable neural interfaces are designed to provide a high spatial and temporal precision control signal implementing high degree of freedom real-time prosthetic systems. The development of a Radio Frequency (RF) wireless neural interface has the potential to expand the number of applications as well as extend the robustness and longevity compared to wired neural interfaces. However, it is well known that RF signal is absorbed by the body and can result in tissue heating. In this work, numerical studies with analytical validations are performed to provide an assessment of power, heating and specific absorption rate (SAR) associated with the wireless RF transmitting within the human head. The receiving antenna on the neural interface is designed with different geometries and modeled at a range of implanted depths within the brain in order to estimate the maximum receiving power without violating SAR and tissue temperature elevation safety regulations. Based on the size of the designed antenna, sets of frequencies between 1 GHz to 4 GHz have been investigated. As expected the simulations demonstrate that longer receiving antennas (dipole) and lower working frequencies result in greater power availability prior to violating SAR regulations. For a 15 mm dipole antenna operating at 1.24 GHz on the surface of the brain, 730 uW of power could be harvested at the Federal Communications Commission (FCC) SAR violation limit. At approximately 5 cm inside the head, this same antenna would receive 190 uW of power prior to violating SAR regulations. Finally, the 3-D bio-heat simulation results show that for all evaluated antennas and frequency combinations we reach FCC SAR limits well before 1 °C. It is clear that powering neural interfaces via RF is possible, but ultra-low power circuit designs combined with advanced simulation will be required to develop a functional antenna that meets all system requirements.

## Introduction

Neural interfaces provide a direct functional interface with the brain to monitor or initiate neural activity. The goal for these devices is to provide real-time control signals for prosthetic devices, study brain function, and/or restore sensory information lost as a result of injury or disease [1]. 

The various classes of neural interfaces can be distinguished by their level of invasiveness (non-invasive and invasive, i.e. intra-cranial) [2]. Non-invasive systems primarily record electroencephalograms (EEGs) from the scalp surface to control computer cursors or other devices. The signals provided by EEGs are typically weak, since the signals are transmitted cross different tissue layers and the background noise also reduces the accuracy of the EEG received signals [3]. Furthermore, EEG-based techniques provide communication channels of limited capacity (20-30 bits/min) [4], limiting the usefulness for prosthetic devices for real-time control. Two other non-invasive technologies that could be considered as neural interfaces are magnetoencephalography (MEG) and functional magnetic resonance imaging (fMRI) [5]. However, both MEG and fMRI technologies require a high field magnetic environment enclosed in a magnetically shielded room, which greatly increases the cost and severely limit their applications. 

The invasive neural interfaces are implanted either on the surface of the brain, or inserted into the cerebral cortex to capture local field potentials and/or action potentials [6-8]. The invasive neural interfaces have the potential to provide the spatial and temporal precision required for implementing real-time prosthetic systems. The utility of neural interfaces have been demonstrated by several labs using non-human primates to control robotic arm movements [9-11] and people with tetraplegia to control a robotic arm [12] and a prosthetic limb [13] . The initial results suggest that neural interfaces implanted in cortex could use spiking activity to restore independence for humans with paralysis [14]. 

Most invasive neural interfaces use wires for power and data transmission. The wires not only limit the utility of neural interfaces, but also increase the likelihood of device failure and clinical risks [15]. Using Radio Frequency (RF) to power and communicate with a neural interface could widely extend the number of applications and increase chronic in-vivo viability. There are several advantages to wireless implementation of neural interfaces: 1) the surgical access can be closed, 2) devices could be distributed across the brain, and 3) it minimizes relative motion between the device and tissue by removing tethering forces. However, RF exposure may result in tissue heating, which is regulated by the Food and Drug Administration (FDA), International Electrotechnical Commission (IEC) and Federal Communications Commission (FCC). In order to comply with these standards, accurate heating effects and RF exposure must be estimated. In addition, it is essential to perform an analysis of electromagnetic power deposition throughout the human head to determine the amount of available power to neural interfaces without violating these limits. Hence, this work focuses on the RF power produced/received by dipole antennas in or on the surface of a human brain and the associated tissue heating. The dipole antenna design was chosen in order to set up a normalized model for the future studies. 

Power deposition analyses have been performed in the design of transcutaneous transmission coils for powering devices (such as cochlear implants), as well as to simulate the effects of external antennas (e.g. cell phones, magnetic resonance imaging probes, and hyperthermia antennas) placed in close proximity to biological tissue [16,17]. Studies have been conducted on the effects of implantable electric devices placed on the retina, cardiac muscle, and other structures within the body [18-26]. However, none of the above studies examine wireless operation inside the brain. 

A miniaturized neuroprosthesis suitable for implantation into the brain was studied by Mojarradi, et al [27], where they measured performance of low power low-noise CMOS preamplifiers. Bashirullah et al. [28] provided a brief overview of developments towards the Florida wireless implantable recording electrode micro systems as well. Harrison et al. [29] presents bench and in vivo experimental results from an integrated circuit designed for wireless implantable neural recording applications demonstrating wireless and inductively powered neural recordings from a cat and non-human primate using a single-chip system (INI3 chip) with a minimal number of off-chip components. None of these studies examine tissue heating increases inside the human brain due to the wireless operation.

Kim et al. studied the thermal impact from the operation of the implanted integrated electrode array (UEA) device [30]. SAR was measured within a human-head-equivalent phantom during operation of the embedded passive wireless neurorecording microsystem [31]. Nevertheless, SAR and temperature changes due to the RF radiation by the wireless RF transmitting antenna haven’t been investigated. Ibrahim et al. provided an initial estimation of the amount of tissue heating under the SAR limitation with the operation of a wireless neural interface device [32]. However, all these calculations were performed in two dimensions (2-D) finite difference time domain (FDTD) method and the peak temperature changes caused by electromagnetic absorption in the head were predicted using the 2-D bio-heat equation. In the 2-D simulation, the simulated head model has to be highly simplified, as well as the structure of the transmit/receive antennas and the integrated implantable chip. Therefore, these models only provide an estimate of heating and SAR. For engineering neural interfaces for human applications, it is critical that we are able to accurately simulate specific 3D antenna structures and chip dimensions. 3-D simulation provides critical data for calculating the transmit power, radiation efficiency and the SAR distributions during device design. The presence of human tissues at high frequencies can affect RF field distribution/intensity/polarization; all of which will impact the allowed power reception under specific SAR guidelines. In conclusion, an elaborate three dimensional (3-D) SAR and temperature study of the implantable neural interface device is needed to accurately model SAR and temperature associated with RF powered neural interface operation.

In this work, we designed a 3-D modeling scheme of the head-neural interface antenna system to study RF power reception and local heating associated with the operation of a wireless implantable neural interface. The dipole antennas were numerically implanted inside of a (1-mm)^3^/19-tissue head model [33-37] at different depths. The study was performed with different antenna lengths at different frequencies. Since FDTD method has great advantage when applied to the human body simulation (relative short computational time and small memory requirements), an in-house 3-D FDTD package was used to calculate the SARs, in conjunction with an accurate excitation/reception algorithm [38]. The FDTD model of the implanted antenna was validated by the analytical formulation on a simplified geometry for uniform dielectric and lossy media. The 3-D bio-heat equation was then used to calculate the temperature changes in the head due to the external antenna. 

## Materials and Methods

### 1: The Numerical Electromagnetic Model

The neural interface is implanted intracranially, including the antenna and all the neural signal processor (spike detection, signal conditioning, RF/DC converter, impedance matching, and analog to digital converter, etc). Since our focus is on the RF power reception by the implanted antenna and the associated tissue heating; the chip structure will be simplified and the antenna performance will be emphasized. An external transmitting antenna is used to transmit power to the implanted receiving antenna within the skull. In our analysis, both the transmitting (outside the head) and receiving antennas (inside the head) were designed as dipole antennas. The dipole antenna was chosen to set up a normalized model for the future studies. 

**Figure 1:**
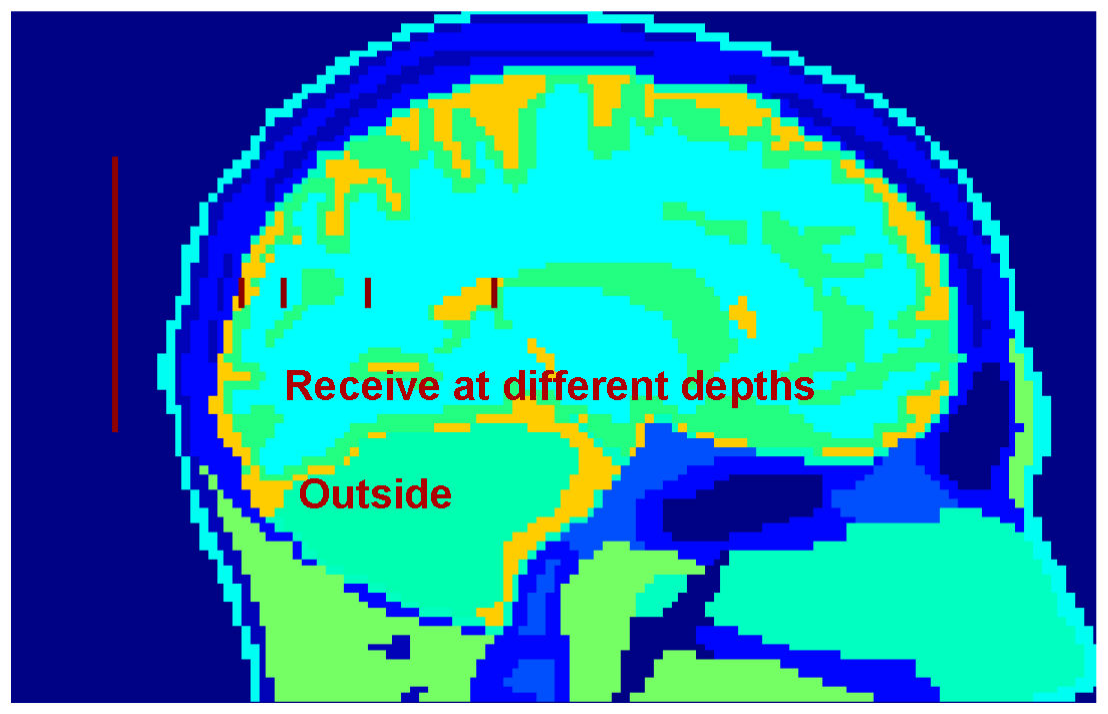
Sagittal view of the human head model. (Lines represent simulated positions of the transmitting/external antenna outside of the head and the implanted neural interfaces at 4 different depths inside the skull.).

The transmitting antenna has a length of 63 mm and is located 10 mm away from the back of the head as shown in Figure 1, and it resonates at a frequency of 2.38GHz in the free space (achieved numerically). The receiving antenna, as a part of the neural interface, is implanted inside the skull. To calculate and analyze the efficiency of power transmission, the receiving antennas were designed with three different lengths (5 mm, 9 mm, and 15 mm) and tested at various depths (0 mm, 10 mm, 30 mm, and 60 mm) inside the brain (the brain surface is normally 20mm from the head surface). The radiation efficiency is in part proportional to the radiation resistance (the real part of the antenna input impedance) [39,40]. Ideally the radius of the wire does not affect the input resistance [40]. Therefore the thickness and width of the wire of the implanted dipole antennas are negligible in this study. The material of the antenna is simulated as the perfect electric conductor (PEC) to model very good conducting materials. The positions of the external antenna and 4 implant depths are illustrated in the sagittal plane of the human head in Figure 1. 

The FDTD grid of the 19-tissue head model developed from 1.5 tesla MR images [33] has a resolution of 1*mm* x 1*mm* x 1*mm*. The FDTD grid of the head-neural interface system has dimensions of 162 x 278 x 200 cells with the spatial resolution of 1mm. The time step is 1.8873 picoseconds to satisfy the FDTD stability criterion. The perfectly matched layers (PML) [41] are used as the absorbing boundary conditions.

### 2: Transmission Line Excitation/Reception and Power Calculations

At the feeding location, the transmitting dipole antenna is excited by a virtual transmission line [42], which is injected with a differentiated Gaussian pulse with sufficient frequency content around the intended operational frequency. The differentiated Gaussian pulse is:

$$G(t)=\frac{1}{T\times 10^{-12}}(t-S\times T\times 10^{-9})\exp{\left(-{\left(\frac{\left(t-S\times T\times 10^{-9}\right)}{T\times 10^{-9}}\right)}^{2}\right)}^{}$$ (1)

**Figure 2:**
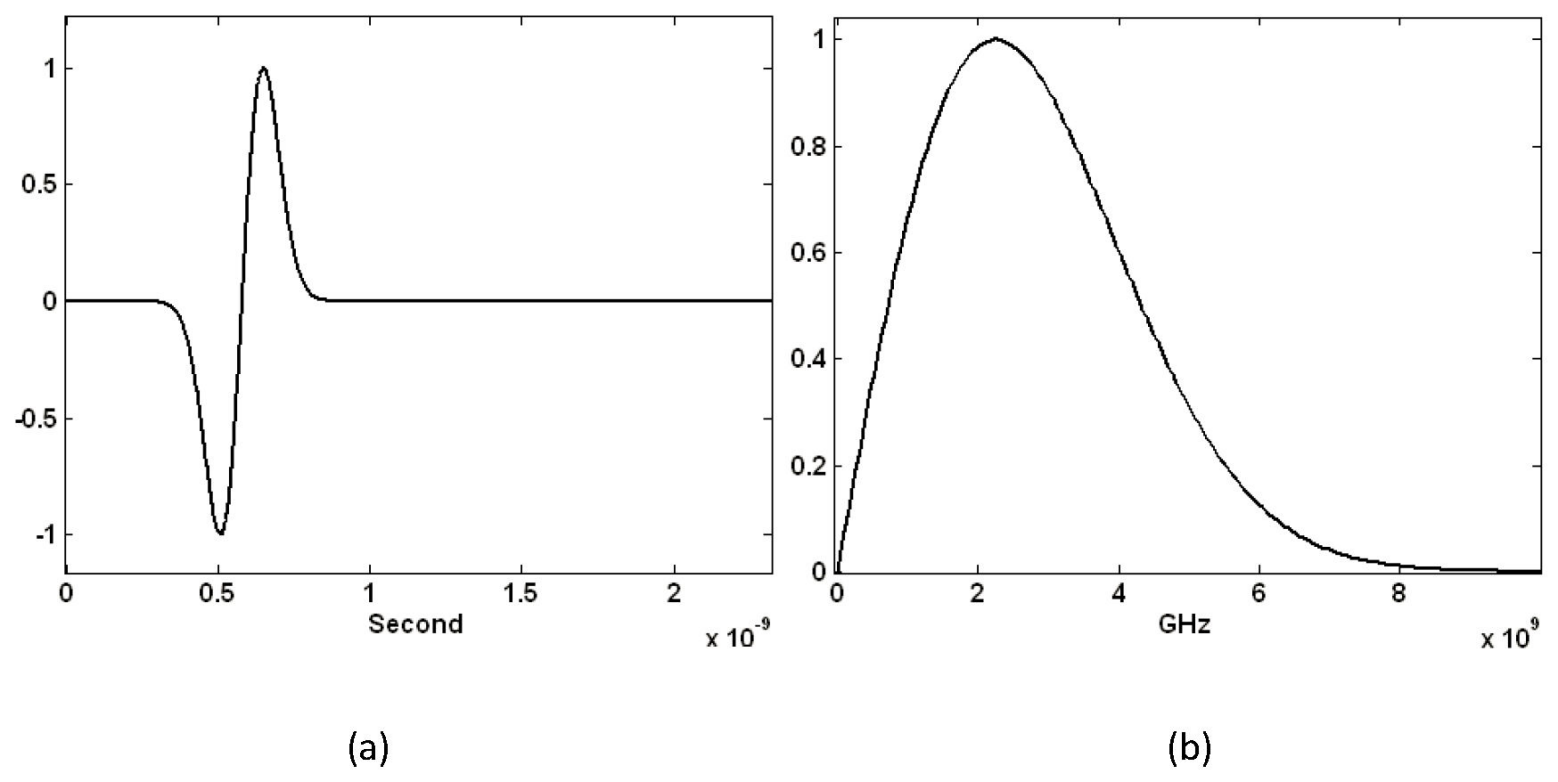
The differentiated Gaussian pulse in (a) time and (b) frequency domains used to power the implanted antenna.

The parameter T affects the pulse-width and the time delay of the pulse. S is a temporal delay parameter. The widely used Medical Implant Communications Service (MICS) frequency band is 401MHz-406MHz [43,44]. Sub-skin-depth implant antenna has been studied around 400 MHz [45]. Recent research reveals that the optimal frequency for the millimeter sized implanted antennas is in the gigahertz range [46,47]. A set of suitable parameters for S (5.8) and T (.1) have been chosen for a wideband spectrum of frequencies ranging from 1GHz to 4GHz according to the lengths of the simulated antennas (5 mm, 9 mm and 15 mm). The differentiated Gaussian pulse in the time domain and the frequency response are shown in Figure 2.

Using an in-house simulation FDTD software that has been experimentally validated in many MRI applications [48-50], a coaxial probe (one dimensional transmission line) feed model is implemented with the standard 3-D FDTD algorithm. This hybrid algorithm is conditionally stable and is subject to continuous adjustment according to the geometry, structure, and properties of the object being simulated. 

A virtual coaxial cable is modeled as a loss-free one-dimensional transmission line [38] connected to the center-fed dipole antennas. The transmission line implementation is used to measure the power radiated by the transmit antenna outside of the head, as well as the power received by the implanted receiving antennas. The power received by each of the implanted antennas is calculated using the following equation:

Prec=12Re[VrecIrec∗] (2)

The load impedance (which will be used to match with the transmission line impedance) *Z*_*L*_ as seen from the transmission line is calculated based on the following equation:

$$\frac{V(z')}{I(z')}=Z_{0}\frac{Z_{L}+jZ_{0}\tan(\beta z')}{Z_{0}+jZ_{L}\tan(\beta z')}$$ (3)

Where *V*(*z’*)*/I*(*z’*) is the ratio of the voltage and current (using frequency domain analysis) at this location, *Z*_*0*_ is the characteristic impedance of the virtual transmission line, *z*’ is the distance between a given point located inside the transmission line and the aperture (interface between the dipole and transmission line) and $\beta =\frac{2\pi }{\lambda }$ is the wave number. 

### 3: Impedance Matching

From circuit theory, a maximum transfer of power from a given voltage source to a load occurs when the load impedance is the complex conjugate of the source impedance [51]. Before calculating the power reception by the implanted antennas, the input impedance and the resonant frequency of a load (composed of antenna, neural interface, human head, and the environment surrounding the head) are computed. After calculating the resonant frequencies and impedances of the load, the characteristic impedance of the transmission line is adjusted to match the load value. The characteristic impedance of the transmission line connected to the external (transmitting) antenna is set to 50 Ohm; while the characteristic impedance of the virtual transmission line connected to the implanted (receiving) antenna is adjusted to the antenna input impedances for the most efficient power reception. 

### 4: The 3-D Bio-Heat Model

Since the wireless RF power produced/received by external and implanted receiving antennas is the focus of this work, temperature changes in the human tissue caused by the RF power deposition in the head with the implanted neural interface antenna due to the radiation from the external transmitting antenna will be considered. After the electromagnetic fields in the human head model are calculated using the FDTD method, the SAR distribution due to the communication between the antennas within the human head model is then computed. The temperature T changes due to the RF field from the external transmitting antenna are calculated using Equation (4) [18]. 

$$\rho C_{p}\frac{\partial T}{\partial t}=K\nabla^{2}T+A_{0}-B(T-T_{b})+\rho SAR$$ (4)

where *C*_*p*_ (J/kg °C) denotes the specific heat (the amount of heat per unit mass required to raise the temperature by one degree Celsius), *K* (J/m s °C) denotes the thermal conductivity (the property of a material that indicates its ability to conduct heat), *A*_*o*_ (J/m^3^ s) denotes the basal metabolic rate (the minimum calorific requirement needed to sustain life in a resting individual), and *B* (J/m^3^ s °C) denotes the blood perfusion coefficient [18,19]. At the boundary between the tissue and air, the following boundary condition is applied [32]:

$$K\frac{\partial T}{\partial n}(x,y,z)=-H_{a}(T_{x,y,z}-T_{a})$$ (5)

where *H*_*a*_ denotes the convective transfer coefficient (a constant with a value of 20 J/m^2^ s °C) [32]. The ambient temperature, *Ta*, is set to 24 °C [18,19]. 

The head model, initially at a uniform 37 °C, is put into a 24 °C environment without RF power deposition (SAR=0) until the equilibrium condition T_0_ is met. A steady state is defined as dT/dt = 2 × 10 ^-7^ °C/s for at least 20 minutes. Then the SAR due to the RF field is inputted in order to calculate the temperature elevations caused by the RF power emitted from the external antenna. The spatial and time steps are 1 mm and 0.0125 second, respectively. The thermal properties of the tissues in the head model can be found in Table 1 [18,32]. 

**Table 1:** Thermal properties for the biological tissues contained in the human head model [18,32].

	Basal Metabolic Rate	Specific Heat	Blood Perfusion Coeff.	Thermal Conductivity
	*A_o_*	*C_p_*	B	K
	[J/(m^3^ s)]	[J/kg °C]	[J/ (m^3^ s °C)]	[J/m s °C]
Air	0	1000	0	0.03
Blood	0	3640	0	0.549
BoneCancellous	590	1300	3300	0.4
BoneCortical	610	1300	3400	0.4
BrainGreyMatter	7100	3700	40000	0.57
BrainWhiteMatter	7100	3600	15925	0.5
Cartilage	1600	3500	9000	0.47
Cerebellum	7100	3700	40000	0.57
CerebroSpinalFluid	0	4200	0	0.62
Cornea	0	4200	0	0.58
Dura	860	2802	4830	0.31
Fat	300	2500	1700	0.25
MucousMembrane	1600	3300	9000	0.43
Muscle	690	3600	2700	0.5
Nerve	7100	3500	40000	0.46
Skin Dry	1620	3500	9100	0.42
Skin Wet	1620	3500	9100	0.42
Tongue^*^	690	3600	2700	0.5
VitreousHumor	0	4200	0	0.6

### Validation

In this section we describe the analytical models of Hertzian dipole antenna immersed in a dielectric and lossy media used to validate our numerical calculations of the implantable antenna. 

**Figure 3:**
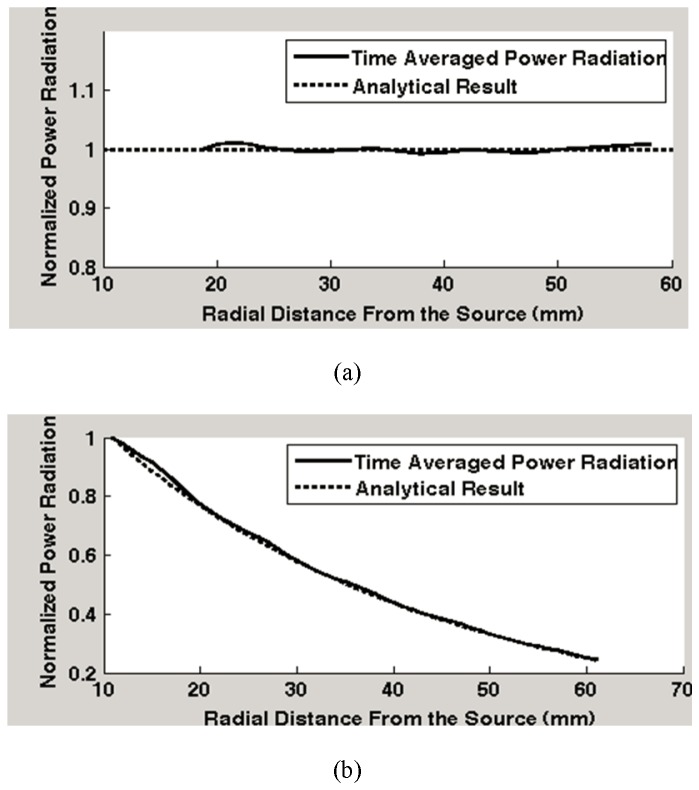
Electromagnetic power radiation in (a) lossless and (b) lossy media. (The solid line represents the FDTD calculated data and dotted line represents the analytical data. The power radiation is normalized and is shown as a function of radial distance from the dipole.).

Two dielectric (lossless/lossy) blocks with cubic shapes are modeled using our FDTD electromagnetic numerical model. Considering the operational frequency of 2.4 GHz, the relative dielectric constant 39.0, and the conductivity 0.39 S/m (average dielectric constant and conductivity in the brain at 2.4 GHz) are used [52]. The resolution of the domain is set 1*mm*×1*mm*×1*mm*and the time step is 1.8873 Pico seconds (similar to that used in our calculations). A coaxial probe feed model is implemented at the center of the calculation domain as a feed point to the Hertzian dipole. Bounded with PMLs, the power radiated from the dipole in the FDTD model propagates similarly as it does in the lossless/lossy medium of infinite extent. After a prescribed number of time steps, the recorded electromagnetic fields in the time domain are calculated at the operational frequency of 2.4 GHz using Fourier transforms. Figure 3 demonstrates the results of the power radiation in (a) lossless (σ = 0, ε = 39.0) and (b) lossy (σ = 0.46, = 39.0) medium. In the simulation of power propagation in a lossless block (Figure 3 (a)), the power radiated through a set of cubic-shaped surfaces enclosing the dipole is calculated as a function of the distance from the dipole. In the case of the lossy medium (Figure 3(b)), instead of the cubical surfaces, the power radiation is computed through a series of spherical enclosures centered on the dipole for comparison with the analytical result (shown later in Equation (6)). The calculations in the spherical surface slightly vary with the radius of the spheres when rectangular cells in the FDTD model are applied to a polar coordinate. Therefore, the power radiation through a sphere enclosure is averaged over three adjacent spherical layers (the resolution of the spherical layers is 1 mm which is the same as that used in the FDTD mode.)

The output from the full wave FDTD model in a lossless medium Figure 3 (a) shows that the total power radiated outwardly measured from a cubic surface is conserved: the simulation result of the time average power radiation over one period ($\lambda =\frac{2\pi }{\beta }$where $\beta =\omega \sqrt{\mu \varepsilon }$ for a lossless medium) minimally changes with propagation (less than 1% difference from the normalized value), which agrees with the energy conservation law [53]. 

According to the power calculation from Equation (6) [54], radiated power of a Hertzian dipole immersed inside a lossy medium is a function of the operational frequency, the radial distance from the source, and the properties of the excitation source and the medium. 

P=Re{∫02π∫0πSR2sinθdθdϕ}=ω3p2μ12π(α2+β2)[2αβR3+4α2βR2+2αβ(α2+β2)R+β(α2+β2)2]e−2αR (6)

where *S* is the complex Poynting’s vector given as $S=\frac{1}{2}E_{\vartheta }H_{\varphi }^{*}$ ; *R* is the radial distance from the source; α and β are the real and imaginary parts of the propagation constant γ given in Equation (7)

$$\gamma =\alpha +j\beta =j\omega \sqrt{\mu \varepsilon }{\left(1+\frac{\sigma }{j\omega \varepsilon }\right)}^{\frac{1}{2}}$$ (7)

According to Equation (7), analytical calculation is performed and the normalized power radiation is plotted as a function of radial distance from the excitation source shown with the dotted line in Figure 3 (b). The simulation results are in excellent agreement with the analytical results. In a lossy medium, the simulation results show that electromagnetic energy decays with its propagation as shown in Figure 3 (b). Similarly, the time average power radiation over one period ($\lambda =\frac{2\pi }{\beta }$ ) from the FDTD simulation clearly predicts the analytical results. 

## Results and Discussion

### 1: Resonant frequencies and input impedances of the Implanted Antennas

The load as seen from the transmission line (composed of antenna, human head, and the environment surrounding the head) is numerically computed by FDTD method. Table 2 lists the resonant frequencies (defined as the frequency at which the implanted antenna input impedance is purely real) and the corresponding input impedance for the three specified antennas at the four specified brain depths. The transmission line connected to the receiving dipole should be adjusted to these impedance values individually in order to maximize power reception. Table 2 demonstrates that implanted dipole antennas with the same length resonate at different frequencies when implanted at various brain depths. Thus the input impedance (at resonance) of the implanted antennas (as defined Equation (3)) varies with the antenna length as well as the position within the human brain indicating that the received near-field RF power maybe impacted by constitutive parameters of the surrounding tissues (table 1).

**Table 2:** Resonant frequencies and input impedances for the dipole antennas implanted at various brain-depths.

Antennas(length)	0mm brain- depth		10mm brain- depth		30mm brain- depth		60mm brain- depth	
	f (GHz)	Z (Ohm)	f (GHz)	Z (Ohm)	f (GHz)	Z (Ohm)	f (GHz)	Z (Ohm)
5 mm	3.39	16.5	3.56	12.0	3.59	12.1	3.01	14.6
9 mm	2.07	22.7	2.16	16.5	2.18	14.6	1.95	16.5
15 mm	1.27	27.1	1.31	23.1	1.37	18.4	1.27	18.3

### 2: Maximum Power Reception without SAR Violations

The SAR safety regulations regarding RF power deposition in the head varies for different applications: the International Electrotechnical Commission (IEC) and the Food and Drug Administration (FDA) limits local SAR <=10 W/kg over every 10 grams of tissue for the heating due to the RF exposure during MRI experiments (normally the frequency is less than 300MHz for human MRI studies). According to FCC safety regulations, the local SAR peak for any 1gm of tissue must be less than or equal to 1.6 W/kg when a human head is exposed to an external radiofrequency field[55]. In this work, the power receptions of the implanted antennas are analyzed based on the FCC SAR safety limit, which covers the frequencies up to 6 GHz.

**Figure 4:**
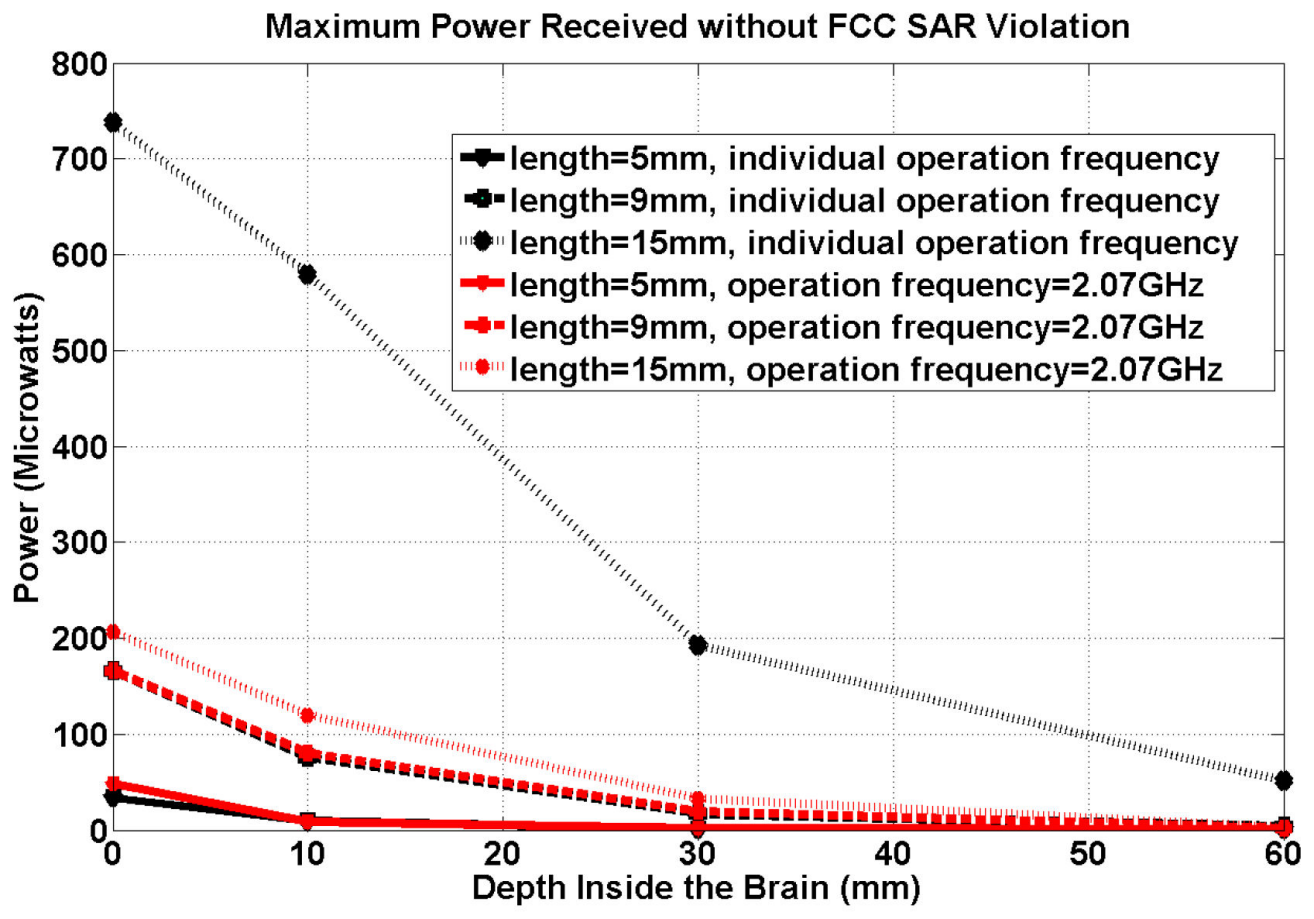
Maximum power reception for all 3 antenna geometries at 2.07 GHz (red) and different frequencies tuned to their individual geometries (black) (maximum power reception at FCC SAR safety limit.).

Figure 4 shows the maximum receiving RF power at the FCC SAR limit for the three dipole antennas at their individual resonant frequencies (shown in Table 2) and at various brain depths. Friis transmission formula indicates that in the far field regime and in lossless mediums, the power received is inversely proportional to the square of the electrical distance between the transmit and receive antennas. Figure 4 demonstrates that the relation between power reception and the implantation depth of the neural interface device does not strictly follow the Friis transmission formula due to 1) the inhomogeneous and lossy environment (human head) and 2) near field effects. Figure 4 also shows that longer antennas receive more power than shorter ones at their individual resonant frequencies. Therefore, the results clearly show that for the shorter dipoles, the available RF power decays with a more rapid rate than the longer dipoles at greater depths inside the brain.

Furthermore, Figure 4 provides the maximum power reception values at the FCC SAR safety limit at 2.07 GHz (the resonant frequency of the 9-mm antenna on the surface of the brain) for the 5-mm, 9-mm and 15-mm antennas implanted at different brain depths. The results show that longer antennas at shallower brain-depths often receive more RF power at the FCC SAR limits even operating at the non-matched/non-resonant frequencies. For example at a specified brain depth, the 15 mm dipole is still the most efficient antenna when compared to the 5-mm and 9-mm antennas even though the operational frequency (2.07 GHz) is 800 MHz away from its resonant frequency (1.27 GHz as shown in table 2.) 

Figure 4 with Table 2 demonstrates that the operating frequency significantly affects the power reception of the implanted antennas: higher frequencies result in less power availability at the SAR limit. The loss in power at higher frequencies is a result of the reduced skin depth; thus, converting much of the RF energy into heat in the superficial tissues. However, the use of lower frequencies can possibly alter the intrinsic impedance of the antenna which can result in significant mismatch with the circuits’ impedances. Therefore, a balanced choice of antenna geometry and operational frequency is crucial. 

Last but not the least, the development of neural interfaces capable of recording from deeper structures may require ultra-low power circuit designs. The antennas’ performances at different operational frequencies in Figure 4 show that the maximum power available before violating the FCC SAR limit for the 15-mm implanted antenna at its resonant frequency will be 190uW or less when the neural interfaces implanted at the brain depths greater than 3cm (or equivalently 5cm inside the head). Assuming a 25% RF/DC conversion efficiency (due to the switching nature of the harvester circuits), the neural interfaces can consume 47.5 uW or less. 

### 3: Temperature Changes

A maximum temperature elevation of less than 1.0 °C is regulated by the FDA government safety guideline [32,56]. In this paper, we evaluated the temperature changes due to the RF radiation by the transmitting antenna. 

**Figure 5:**
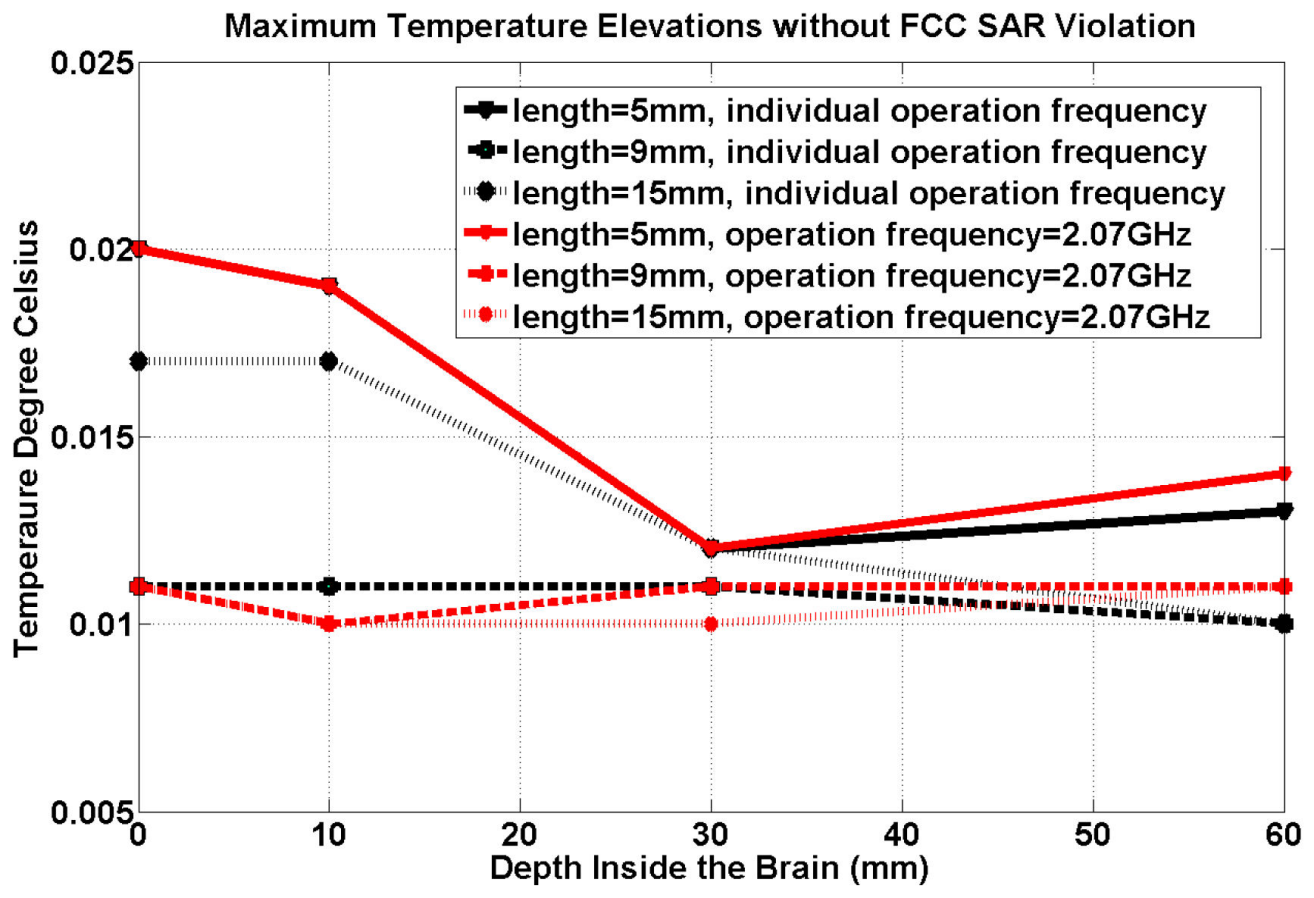
Maximum temperature elevation for all 3 antenna geometries at 2.07 GHz (red) and frequencies tuned to their individual geometries (black) at FCC SAR safety limit.

Figure 5 shows the maximum temperature elevations due to the RF radiation by the transmitting antenna when the receiving antenna is implanted at various depths. It shows that a maximum of 1.6 W/kg per 1gm SAR results in a temperature increase that is less than or equal to 0.02 °C for all cases. At the same operational frequency of 2.07GHz, the maximum temperature elevations for an antenna at various brain-depths are similar. This could be explained based on the Equation 4: the temperature changes due to the RF radiation of the external antenna mainly depend on the SAR distribution; since the maximum SAR is limited to the same value (1.6W/kg averaged over every gram of tissue), the increased temperature is expected to be very similar. Furthermore, because of the thermal diffusion was considered in this 3D simulation, small-scale variations in SAR do not necessarily lead to biologically significant variations in temperature [57]. 

**Figure 6:**
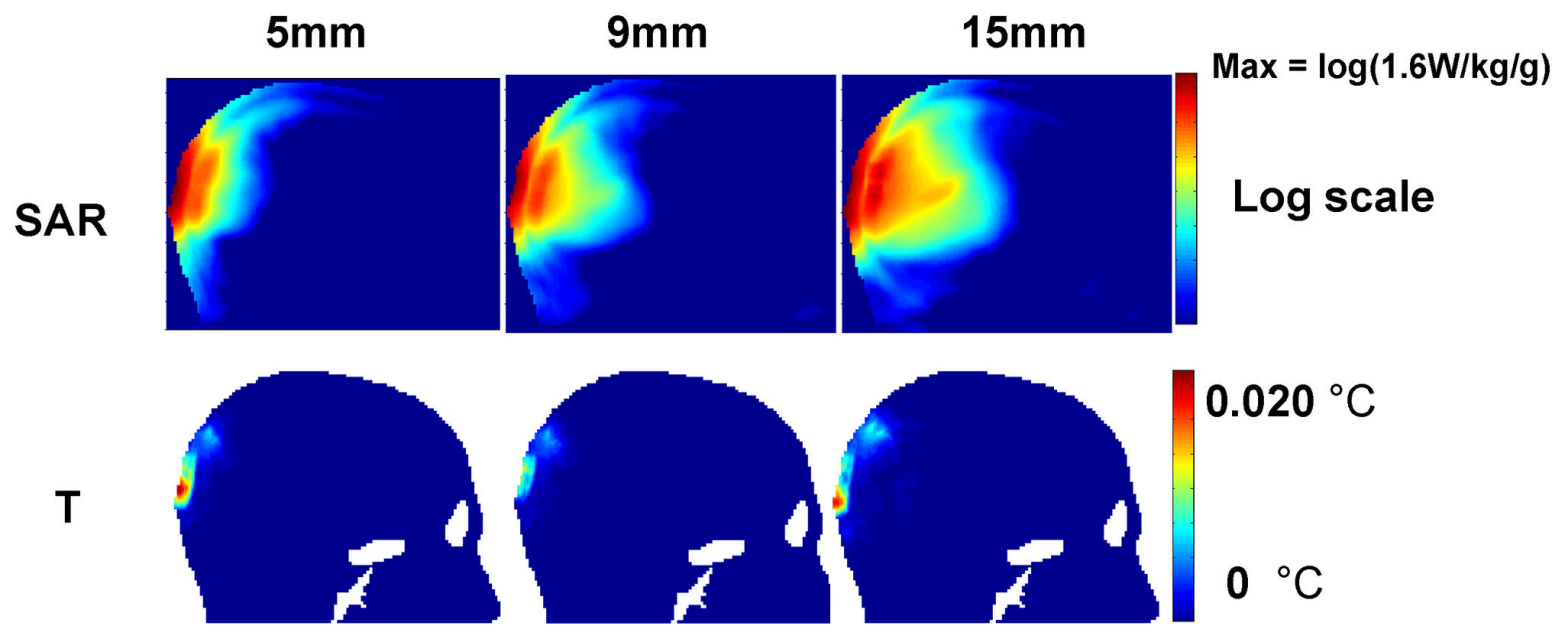
Logarithmic SAR and temperature (T) distributions for the 3 antennas positioned at 0-mm brain depth.

Figure 6 provides a set of examples of the logarithmic SAR and temperature distributions for the three antennas at 0 mm brain-depth. The top row shows the logarithmic SAR distributions for the 5-mm, 9-mm and 15-mm antennas (each operating at its resonant frequency). Comparing the results in the top row, the deposited RF power extends deeper into the brain at the lower operating frequencies (longer antenna operating frequency) than higher frequencies (shorter antenna operating frequency) with the same SAR peak (1.6 W/kg averaged over 1gm of the tissue), this is because the power is decaying faster at higher frequency than at the lower frequency). The bottom row of Figure 6 shows the corresponding temperature elevations caused by SARs shown in the top row. The surface of the head nearest the transmitting antenna experiences the greatest temperature rise. This is expected, since the SAR and temperature peaks calculated in this section are due to the transmitting antenna rather than the implanted receiving antenna. Comparing the temperature distributions and the SAR distributions, the temperature distributions do not always correlate with SAR distributions; therefore predicting the locations of the hot spots (where highest temperature rise occurs) based on the SAR distribution alone can be misleading. This issue has been discussed in previous works involving high frequency electromagnetic field biological tissue interactions [58,59]. 

## Conclusion

The maximum power receptions (at the FCC SAR limit) by the implanted antennas were calculated for the three different dipole antenna geometries. The results demonstrate that a longer-length implanted antennas (when dipole antennas are utilized) with lower operational frequencies (not necessarily the antennas resonance frequencies) and shallower implantation depth will maximize the RF power reception prior to violating the safety limits. However, the use of lower frequencies can possibly alter the intrinsic impedance of the antenna which can result in significant mismatch with the circuits’ impedances. Therefore, a balanced choice of antenna geometry and operational frequency is crucial. The corresponding temperature elevations calculated using 3-D bio-heat simulations show that for the antennas and frequencies evaluated the highest temperature increase was less than 1 °C. 

The development of neural interfaces capable of recording from deeper structures may require ultra-low power circuit designs. Neural interfaces must be capable of operating with less than 47.5 uW of power when implanted at depths greater than 3 cm inside the brain (or equivalent 5 cm inside the head) based on the results in this work. Our current designs of implantable neural interface sensors consume about 35 uW [60]

For new applications that do not possess their own specific SAR regulation such as the case with measuring neural activity with wireless (RF powered) microneural interface (the topic of this work), new SAR limits may be determined using factors specific to the application of interest including: 1) Findings from experimental studies/numerical methods and/or 2) More complete understanding of long term consequences of exposure to electromagnetic fields. 

The maximum allowed received power will then change based on new SAR limits.  For instance, we have calculated the maximum power reception when IEC/FDA SAR regulation is applied and the results shows that the allowed power reception will be almost ten times of the power received when FCC SAR limit is applied: the maximum power reception for receiving antennas operating at their resonant frequencies (shown in Table 2) and implanted at the surface of the brain using IEC/FDA SAR safety regulations are 393.02 µW, 1996.30 µW  and 7667.91 µW for 5 mm , 9 mm  and 15 mm antenna respectively.  The temperature elevations for these cases are 0.70 °C, 0.83 °C and 0.84 °C for the 5 mm, 9 mm and 15 mm antennas respectively.  This compares to power reception values of 33.25 µW, 166.03 µW, and 737.69 µW and temperature elevation values of 0.02 °C, 0.011 °C, and 0.017 °C when the FCC SAR limit is applied. 
